# Current Status of Echinoderm Genome Analysis - What do we Know?

**DOI:** 10.2174/138920212799860643

**Published:** 2012-04

**Authors:** Mariko Kondo, Koji Akasaka

**Affiliations:** Misaki Marine Biological Station, Graduate School of Science, and Center for Marine Biology, The University of Tokyo, Japan

**Keywords:** Echinoderm, genome, sea urchin.

## Abstract

Echinoderms have long served as model organisms for a variety of biological research, especially in the field of developmental biology. Although the genome of the purple sea urchin Strongylocentrotus purpuratus has been sequenced, it is the only echinoderm whose whole genome sequence has been reported. Nevertheless, data is rapidly accumulating on the chromosomes and genomic sequences of all five classes of echinoderms, including the mitochondrial genomes and Hox genes. This blossoming new data will be essential for estimating the phylogenetic relationships among echinoderms, and also to examine the underlying mechanisms by which the diverse morphologies of echinoderms have arisen.

## INTRODUCTION

**Fig. (1):**
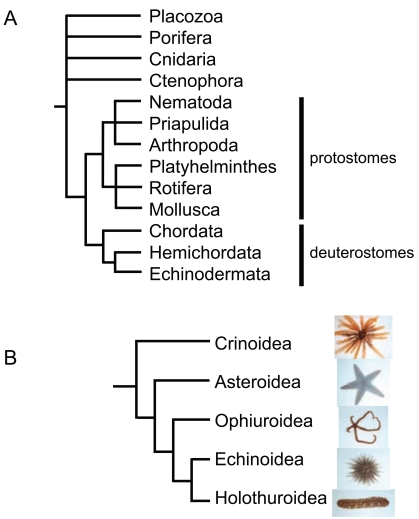
Phylogenetic relationships among animals (**A**) and echinoderms (**B**). (**A**) Echinoderms (phylum Echinodermata) are placed in the group of deuterostomes, with other species of the phyla Chordata (vertebrates, tunicates etc.) and Hemichordata. Not all phyla are included in this tree. Placozoa, Porifera, Cnidaria and Ctenophora are more basal in animals, although their phylogenetical positions are under debate. (**B**) The evolutionary tree based on fossil records, reconstructed from [12]. Extant echinoderms species from each class are shown as examples: from top to bottom, *Oxycomanthus japonicus* (Crinoidea), *Astropecten scoparius* (Asteroidea), *Ophiothrix nereidina* (Ophiuroidea), *Temnopleurus reevesii* (Echinoidea), and *Pentacta australis armatus* (Holothuroidea).

Echinoderms are deuterostome invertebrate animals, phylogenetically most closely related to hemichordates and to chordates. They are exclusively marine-living, with a wide range of habitats from the deep sea to the intertidal region. Echinoderms are characterized with spiny skin, from where their name originates, and their internal skeletons are composed of mesodermally derived calcareous ossicles. These animals show other distinctive characteristics in their morphology that fascinate biologists, which include a radial adult body with pentamerous symmetry, and a water vascular system. Due to their unique body plan and position in the phylogenetic tree (Fig. **1A**), echinoderms, especially sea urchin, starfish and sea cucumbers, have served as model animals in research because of their high availability and ease of maintaining in the laboratory. 

Crucial processes of the cell cycle were first identified in the sea urchin [1] including the identification of the cyclin proteins that regulate cell cycle progression. Phagocytosis of microbes was first demonstrated in sea stars by Mechnikov in the 1880s. Both of these findings have been awarded Nobel Prizes. Other very important discoveries using echinoderms, in particular sea urchins, are, for example, the fusion of egg and sperm nuclei in fertilization [2], or the identification of chromosomes that carry hereditary information (reviewed in [3]), both of which have been reported over a century ago, and the more recent analyses of the gene regulatory network (reviewed in [4]). Echinoderms are now used in broader research fields, such as evolutionary studies (including evolutionary developmental biology) and marine ecology. Since the lifespan of some sea urchin species is known to be very long, these animals also are considered model animals for aging research. For example, it is estimated that *Strongylocentrotus franciscanus* lives up to 200 years [5], and *Echinometra lucunter lucunter* [6] and *Sterechinus antarcticus* up to 75 years [7]. Regeneration of echinoderms has also been a long topic of research, with important scientific papers dating back to the 19th century (see references in [8]). 

Ever since the complete sequencing of the fly and human genomes was accomplished, genomes of many different animal species have been analyzed. This is in great part due to the advancements in sequencing methodologies and the decrease in cost. According to the genome resource of NCBI (http://www.ncbi.nlm.nih.gov/sites/genome/), as of May 2011, 416 genome sequencing projects (entries) are either completed, assembled, or in progress (Table **1**). As one might easily guess, chordates are the most sequenced group of animals, with about 200 projects working on mammals. In addition, compared to chordates, with the exception of arthropods, other animals including echinoderms are not well investigated, and the sea urchin *Strongylocentrotus purpuratus* is the only fully sequenced echinoderm [9]. 

The primary reason why the whole genome of the sea urchin was sequenced is because the echinoderm embryo is a useful model system [9]. A wide variety of development occurs in echinoderms; this includes direct or indirect (through a feeding larval stage) development, and a variety of larval structures such as the pluteus, auricularia, doliolaria, etc., and some species even go through a combination of these stages (reviewed in [10]). As mentioned above, echinoderms possess synapomorphic characters that are distinctively different from other animal groups, notably, pentamerous radial symmetry, but embryos/larvae show bilateral symmetry, and when and how pentamerous body structures arise differ among classes. For example, the pentameral body plan of sea urchins is formed in the adult rudiment that forms in the later larval stage of development. Sea cucumber, which is a direct developer, developed *via *metamorphosis from auricularia to doliolaria, in which pentameral symmetry of the water-vascular system is formed. Feather stars also do not form adult rudiments, and a five-fold symmetry is first observed in the calyx (the crown-like part) of cystidean larva, which has settled and developed a stalk. Thus, morphological divergence within the phylum is well worth studying, but in reality, the genes and genomic regions involved in the mechanisms that are fundamental to such phenomena are unknown and probably difficult to identify. Therefore, sequencing the genome should be an aid to seeking functional genes in the genome and to unfold that makes these animals so similar to each other but divergent, both between echinoderm classes and other deuterostome phyla.

This review of the echinoderm genome aims to provide an overview of the current status of genome research as of 2011, and to introduce several topics that were found from the analysis of the sea urchin genome. Furthermore, we intend to introduce broad representatives of animals of the phylum Echinodermata, which are used in molecular studies.

## PHYLOGENY OF ECHINODERMS

Five taxonomic classes comprise the phylum Echinodermata, namely Crinoidea (sea lilies and feather stars), Asteroidea (starfish or sea stars), Ophiuroidea (brittle stars), Echinoidea (sea urchins), and Holothuroidea (sea cucumbers). Crinoidea is the only extant class that constitutes the subphylum Pelmatozoa, which left a robust fossil record during the Pelmatozoa. Extant crinoids are largely divided into two groups, feather stars (or comatulids) and sea lilies. The former are stalkless and vagile, whereas the latter are stalked and basically sessile. Other echinoderms belong to the free-living subphylum Eleutherozoa. 

**Fig. (2):**
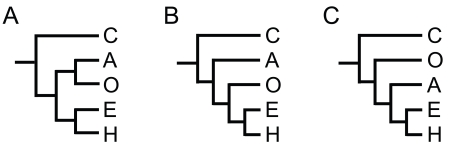
Phylogenetic relationships among echinoderms. Three trees (**A-C**) of echinoderm classes were deduced from morphological and/or molecular data [14]. The position of Crinoidea being most basal is supported, but topologies are not conclusive, as tree (**C**) is not supported by morphological data, and the position of Ophiuroidea is still debated. Abbreviations: A, Asteroidea, C, Crinoidea, E, Echinoidea, H, Holothuroidea, O, Ophiuroidea.

The relationship among the five extant classes of echinoderms has been an issue that has long been debated. Phylogeny has been estimated primarily using cladistic methodology, and more recently with molecular data (reviewed in [11]). Fossil records indicate that *Camptostroma*, the common ancestor of the extant echinoderms (crown-group echinoderms) appeared during the early Cambrian [12], from which pelmatozoan echinoderms diverged. The basic body plan of eleutherozoan fossils appear about 475 to 480 Ma as asteroids, then the lineage of ophiuroids diverge, and the echinoids and holothurians occur about 450 Ma (reviewed in [13]) (Fig. **1B**). This suggests that crinoids and allies are the most ancient representative of extant echinoderms. Littlewood *et al*. [14] have published a phylogenetic study that included larval and adult morphologies as well as molecular data of 18S and 28S rDNA sequences. This study proposed three phylogenetic relationships (Fig. **2A-C**), two of which (Fig. **2A** and **B**) are well supported by morphology. In other studies, using mitochondrial protein-coding genes such as cytochrome c oxidase (COI, COII, and COIII) and cytochrome b (cob), or 18S rDNA, phylogenetic trees have been constructed [15-21]. Although monophyly of the classes is supported, construction of phylogenetic trees with different genes, taxa and method of analysis often result in contradictory topologies. For example, phylogenetic analysis using mitochondrial protein-encoding genes resulted in the conclusion that ophiuroids have long branches and are the most basal of the echinoderms [16, 17]. Substitution rates appear to differ within the echinoderms, and particularly, ophiuroid species seem to have accumulated many substitutions which are presumably caused by more rapid evolution, and indeed are not the most ancient of echinoderms. 

Partial or complete mitochondrial genome sequences of species from all the 5 extant classes of echinoderms have been reported and used for solving the phylogeny of echinoderms. The echinoderm mitochondrial DNA is approximately 16 kb long, and those so far analyzed have a region with generally conserved gene order in all classes. There are some exceptions; for example, a 4.6-kb inversion is seen between the mitochondrial genomes of starfish and sea urchins [22, 23], which consist of a part of the tRNA cluster, 16S (large) ribosomal RNA gene and the NADH dehydrogenase subunit 1 and 2 (ND1 and ND2) genes. Thus, gene rearrangement comparisons might be useful to include in the analyses of the relationship between classes [15-20, 24]. It was additionally shown that from similarity in mitochondrial gene order, starfishes are grouped with brittle stars, and sea urchins with sea cucumbers [24]. In the study by Perseke *et al.* [15], a hypothetical crinoid gene order was proposed. However, overall, mitochondrial gene order gives only inconclusive answers. As some studies admit, there seem to be limitations to using mitochondrial gene order rearrangements as a global phylogenetic tool in the case of echinoderms. This is due to the occurrence of extensive rearrangements in crinoids and especially in ophiuroids [15, 17]. It is considered that accumulation of molecular information from a wider variety of taxa is required for solving the echinoderm phylogeny. The study by Janies (2001) [25] used data from increased numbers of taxa for the analysis; however, a recent study showed that tree topologies are more dependent on the methods used for reconstruction [26].

In conclusion, the phylogenetic relationship of echinoderms is not clear-cut, and it is difficult to state which is the most favored relationship. However, the “total evidence tree” proposed by Littlewood *et al. *(1997) [14] (Fig. **2B**) is in some studies mentioned for the evaluation and comparison whether a newly reconstructed tree agrees or disagrees with it (for example, references [19, 20]). Therefore, it may be considered that this tree is the most favored one. 

Whole-genome sequencing provides us with the opportunity to conduct phylogenomic analyses (reviewed in [27]). Genomic data may be analyzed with two phylogenomic reconstruction methods, that is, sequence-based methods and methods based on whole-genome features, and also by studying other characters in the genome, such as insertions and deletions, retroposon integrations and gene fissions and fusions. Phylogenomics have been proven to clarify phylogenies of several groups of organisms, but it has also been shown that the current methodologies do not always give correct solutions [27]. Therefore, since the whole genome sequencing of many echinoderm species has begun, we may be able to obtain a better understanding of echinoderm phylogeny, or on the contrary, may have to face more puzzles. 

## CHROMOSOMES AND GENOME SIZES OF ECHINODERMS

The chromosomes of echinoderms have not been studied from the genomic or genetic point of view, although chromosome numbers have been reported since the end of the 19th century (see references in [28]). Echinoderm chromosomes are cytogenetically not easy to work with. They are relatively small and tightly clustered, and in addition, obtaining mitotically active tissue from organisms is difficult. Therefore, various studies had yielded different chromosome numbers for the same species. Recent studies from all five classes of echinoderms (for example [28-33]), report that the majority of echinoderms possess between 36 and 46 chromosomes (diploid). Heteromorphic chromosomes were observed in mitotic figures from about half of the specimens of the sea urchins *Paracentrotus lividus* [31], *S. purpuratus* and *Strongylocentrotus droebachiensis* [30], and starfish *Asterina pectinifera* [33]. These chromosomes could be sex chromosomes. Similarly, difference in karyotypes between sexes and evidence for sex chromosomes have been observed in other marine invertebrates [34-36]. These findings support the existence of chromosomal sex-determining systems in different lineages. Further investigations using different staining methods may reveal other echinoderm sex chromosomes and sex-determining systems. 

Telomere analysis has also been accomplished in echinoderms including sea urchin telomere structure [37], telomere length and telomerase activity [38]. (TTAGGG)_n_ was identified as the telomeric sequence of *S. purpuratus* by Southern hybridization [37], and this sequence could be found as repeats in the *S. purpuratus* genome sequence (Trace Archive of NCBI, http://www.ncbi.nlm.nih.gov/). This sequence is the same for vertebrates [39] and an urochordate [40]. Telomere length shortening is normally an indicator for aging. However, experimental results from the sea urchin species *Lytechinus variegates* (estimated lifespan of 3-4 years), *S. fransciscanus* (over 100 years) and *E. l. lucunter* (over 10 years) indicated telomerase activity from embryonic stages to adult and no age-associated telomere shortening [5, 6, 38]. 

Fluorescent in situ hybridization (FISH) of chromosomes have been performed for the sea urchin species *P. lividus* [41-43], *S. purpuratus* and *S. droebachiensis* [30], and the crinoid, *Oxycomanthus japonicus* (Ikuta and Saiga, unpublished). Using FISH, locations of ribosomal genes and single copy genes such as brachyury [30] and Hox genes (Ikuta and Saiga, unpublished) have been analyzed. This technique will allow gene maps to be developed, and will facilitate genomic comparisons of synteny between species.

On the other hand, a genetic linkage map of the sea cucumber *Apostichopus japonicus* has been produced based on AFLP (amplified fragment length polymorphism) and microsatellite markers, identifying 20 linkage groups which correspond to the number of haploid chromosomes [44]. Genetic linkage maps are necessary to map phenotypes and QTL (quantitative trait loci), but generally requires collection of markers and breeding of animals. 

Cellular DNA contents have been estimated [45-47] for species from all echinoderm classes except for crinoids. The amount of DNA per haploid ranges from 0.54 pg in the starfish *Dermasterias imbricata *to 4.4 pg in the sea cucumber *Thyonella gemmata*. Thus, the genome size differs approximately 8 fold, from 500 Mb to 4 Gb*.* *S. purpuratus* was estimated to have 0.89 pg of DNA per haploid cell with the standard deviation within +/- 5% [47], which is roughly 800 Mb. As mentioned later in this review, this value matches very well with the sequenced genome size of *S. purpuratus*.

## SEQUENCING OF THE SEA URCHIN GENOME

Sequencing of an entire genome allows the direct comparison of different organisms at the molecular level. The properties that underlie the differences between animal groups or species, or the shared characteristics among a group of animals should undoubtty be present in the genome. So far, the only echinoderm species that has been sequenced is the purple sea urchin, *S. purpuratus* [9]. 

Many genome resources were developed in the process [48]. Sequencing procedures of the *S. purpuratus* genome [49] may be summarized as follows: DNA was extracted from a single male, from which whole genome shotgun (WGS) libraries and BAC libraries were constructed. The BAC clones were used for end-sequencing and to construct a fingerprint map and tiling path. Sequencing was performed to 6-fold with WGS and BAC paired end-sequencing, and 2-fold with tiling path BAC sequencing. Combined, a high-quality draft with 8-fold coverage was generated. The assembly was 814 Mb in size, and is in good agreement with the previously estimated genome size of 800 Mb +/- 5% [47]. The sequence is available and accessible at NCBI Mapviewer (http://www.ncbi.nlm.nih.gov/mapview/) and in SpBase (http://spbase.org/) [50]. The total number of predicted gene models was 28,944, but considering redundancy, pseudogenes and estimates of unrepresented genes (by transcription data obtained from whole-genome tiling microarray analysis), the gene number is estimated to be about 23,300 [9], which is similar to the number in vertebrates. Over 9,000 gene models were manually curated, and protein domains or motifs were identified to classify the protein models and compared to other model species, such as mouse *Mus musculus*, tunicate *Ciona intestinalis*, fruitfly *Drosophila melanogaster*, nematode *Caenorhabditis elegans* etc. The results are summarized in the studies by the Sea Urchin Genome Sequencing Consortium [9] and Materna *et al*. [51]. Reciprocal BLAST match searches were performed to count the strict orthologous protein sets between species. This analysis showed a higher number of sea urchin proteins orthologous to other deuterostome proteins (out of 28,944 sea urchin sequences, 7,021, 7,077, and 6,366 to mouse, human and ascidian, respectively) in comparison to the protostomes (5,344 and 4,475 to fruit fly and to nematode, respectively). Interestingly, though the cnidarian *Nematostella vectensis* and sea urchin are considered phylogenetically distant, they had 7,331 matches, which is a number comparable to that between sea urchin and mammals. 

Approximately, one third of the 50 most abundant gene model groups identified in sea urchin were not included in the top 50 of other representative genomes, indicating expansions in gene models that are specific to sea urchin. The toll-interleukin-1 receptors (TIR) and the scavenger receptor, involved in innate immunity [52], DEATH-like domains and NACHT domains, involved in apoptosis and cell death regulation [53] are among the most expanded domains. Another example is the expansion of quinoprotein amine dehydrogenase domain containing proteins. Echinoderms are characterized by quinone containing pigments and since quinones are redox cofactors for enzymatic activity of quinoproteins, the expansion of this family may be explained by the use of these enzymes in pigment synthesis. Therefore, this expansion is likely to be found in other echinoderms. 

The sea urchin shares about 4000 domains with other bilaterian gene models. In turn, there are 1375 domains from other bilaterian gene models that are not found in the sea urchin genome. For example, the insect specific cuticle protein and the tetrapod vertebrate Krüppel-associated box (KRAB) domain are missing. These missing domains may be unique to certain lineages. Others that seem unlikely to be lineage specific, such as Peptide M, neutral zinc metallopeptidases, zinc-binding site, and serine/threonine protein kinase, active site are found in *M. musculus*, *D. melanogaster* and *C. elegans*, but not in *S. purpuratus* [51]. The missing domains may be accounted by the limitation in search methods. In this case, sea urchin amino acid sequences may have not been conserved well enough to be recognized with current Hidden-Markov models that were built from seed sequences without sea urchin sequences [51]. Different studies on gene family identifications employ various computational programs and gene models, and probably depending on the methods used to assess homologies, the predictions differ between them. Therefore, the gene content cannot be described by domain search alone. For example, though the sea urchin exhibit high sensitivity to environmental stimuli, no gene models assigned to the mouse olfactory receptor or several other members of G-protein coupled receptors (GPCRs) used for chemosensory function in other animals was present in the sea urchin genome [51]. It is acceptable that the mouse olfactory receptor is not found in sea urchin, since olfactory receptors of vertebrates are considered to have arisen by multiple gene duplication events. Meanwhile, an investigation focusing on GPCRs identified 979 rhodopsin-type GPCRs in the genome [54]. Therefore, it seems that chemosensory receptors of the sea urchin are encoded by the genes containing this expanded rhodopsin-like GPCR superfamily [54].

Of the large amount of genes identified from the genome, genes involved in two properties shared between echinoderms and vertebrates, namely immunology and biomineralization, are introduced in the following sections. 

## IMMUNE GENES OF THE SEA URCHIN 

The sea urchin genome provides material to broaden our understanding of the evolution of the immune system. The studies by the Rast group [52, 55] explored in depth the genes related to the immune system. From the analyses of sequence database searches and domain structure, they identified over 1000 gene models with relevance to immunity and other blood cell functions in the sea urchin genome. As mentioned earlier, prominent expansions of three classes of innate immune recognition proteins were found, namely of the Toll-like receptors (TLR), the NACHT and leucine-rich repeat containing proteins (NLR), and the multidomain scavenger receptor cysteine-rich proteins (SRCR). Sequence divergence is seen in the ectodomain of LRR (leucine-rich repeat) motifs, which is speculated to cause diversification of immune recognition specificity [52]. Judging from the characteristics of this gene family, this expanded family seems the result of recent duplications and diversifications. Scavenger receptor proteins are often expressed on macrophages and function in innate immunity. There are 218 gene models with a total of 1905 SRCR domains. Sea urchin coelomocytes express SRCR genes and therefore, the expansion of the genes may reflect immune diversity.

In addition, Ig domain-coding genes were found, encoding putative transmembrane receptors with immunoglobulin variable-type (V) domains similar to those used in adaptive immunity of jawed vertebrates [52, 55]. V-type immunoglobulin is found not only in chordates, but also in arthropods and bacteria. Putative transmembrane receptors with IgV set-IgC1 set ectodomains are found among the sea urchin genes, and these show similarity to vertebrate Ig/TCR/MHC. These gene models could not be found in the sea urchin genome by computational searches described in Materna *et al*. (2006) [51], due to low similarity. The rearrangement of immunoglobulin and T-cell antigen receptor gene families in adaptive immunity is mediated by the activity of proteins including Recombination Activating Gene (RAG) 1, RAG2 and terminal deoxynucleotide transferase (TdT). Again, homologues of these genes were also found in sea urchin, although there has been no evidence for V(D)J recombination. Rag1 and Rag2 genes do not exist in the sequenced *C. intestinalis* genome, and were known only to be present in the genome of jawed vertebrates, arranged in a cluster. The homologous gene pair was found in the sea urchin genome, and furthermore, linked to each other [56]. These findings strongly suggest that the Rag1 and Rag2-like genes were present in the ancestral deuterostome, and the function of these gene products in adaptive immunity was acquired later, in an early jawed-vertebrate [56]. The existence of other immune factors previously found only in chordates or vertebrates, such as interleukin (IL)-1 receptor, IL-17, PU.1/SpiB/SpiC subfamily of Ets transcription factor [57], as well as the above-mentioned NLR proteins, clearly suggest that these were present in the common ancestor of echinoderms and chordates. 

## BIOMINERALIZATION GENES OF THE SEA URCHIN

Echinoderms have characteristic internal skeletons. It is generally accepted that the extensive biomineralized structures of echinoderms and vertebrates have independently arisen. Analysis of genes involved in biomineralization shows that spicule matrix proteins, which are secreted proteins contained within the spicule, and MSP130 proteins, which are the cell surface proteins expressed in skeletal forming cells of the embryo (primary mesenchymal cells), both form small families that are echinoderm specific [58]. In turn, many of the vertebrate proteins that mediate mineral deposition, such as secreted calcium-binding phosphoproteins, do not have counterparts in the sea urchin genome. Other genes that are involved in earlier events in skeletogenesis, such as collagen and metalloproteinases, seem to be shared between the sea urchin and vertebrates. Therefore, the cellular and molecular processes associated with skeletogenesis may have existed in the common ancestor of echinoderms and vertebrates, but after the divergence of echinoderms and vertebrates, each lineage has evolved independently distinct proteins with similar biochemical properties to facilitate biomineralization. Several genes were found in the sea urchin genome that are also present in non-chordate organisms. Two SPARC (osteonectin)-related genes, Sp-osteonectin and Sp-SPARC-like and a calcium-sensing receptor (Casr) gene were among such genes, and homologues are present in vertebrates and protostomes (such as *D. melanogaster*, Artemia and *C. elegans*). Casr is implicated in osteoblast differentiation in mammals. Additionally, an ASPIC (acidic secreted protein in cartilage) domain exists in vertebrates, sea urchin and cyanobacteria, but not in Ciona or Amphioxus. So far, there are no reports of the comparative analysis among a wide group of organisms, but it would be very interesting to find out whether there are processes in biomineralization that are shared in both protostomes and deuterostomes.

## HOX/PARAHOX GENES AND THE HOX CLUSTER

Hox transcription factors are considered to have a crucial role in specifying the anterior-posterior identity of the body during development and are of great interest to explain why echinoderms have unique morphologies. They are evolutionarily highly conserved patterning genes which arose after the divergence of ctenophores from the rest of the metazoans, and they are found in cnidarians and bilaterians [59]. The Hox genes of the fruit fly Drosophila and mouse were found to be related to each other in terms of organization and expression [60, 61] lead to the idea that all other animals should also have similar Hox genes and organization. The generally accepted features of Hox genes are that they are arranged in a cluster on a chromosome, that a correlation exists between the order of genes on the chromosome and the position of expression along the anterior-posterior axis of the developing embryo, and that they share a correlation between gene position and the temporal order of expression. These correlations in expression are called “colinearity”. As Arnone *et al.* [62] pointed out, the term “colinearity” is also used considering the primitive cluster position based on orthologous relationships of the genes, without knowledge of the actual position of genes within a cluster. Since axis formation of echinoderms is quite puzzling, the gene repertoire and genomic organization of Hox genes of echinoderms are fascinating and important topics, and Hox genes are one of the most intensively studied gene groups and genome regions in echinoderms. In addition, since Hox genes are highly conserved and have been characterized in a wide range of organisms, this region is used in genome evolution studies.

Hox genes have been considered to influence morphological evolution through, to name a few instances, gain or loss of Hox gene family members, change in expression patterns, and change in regulatory interactions between Hox proteins and their targets [63]. Therefore, it is easy to suppose that there is a link between modification in Hox genes or the Hox gene cluster organization and the body plan of echinoderms, although a direct correlation between Hox genes and the formation of a pentameral body may not exist, considering reported Hox expression patterns. 

Hox gene cloning from echinoderms has been reported many times, although with a variation in gene components and numbers among taxa: the sea urchin species *S. purpuratus* [64-68], *Tripneustes gratilla* [69-71], *P. lividus* [72], *Hemicentrotus pulcherrimus* [73], *Holopneustes purpurescens* [74], *L. variegatus* [65], *Heliocidaris erythrogramma* [75, 76], *Heliocidaris tuberculata* [76], *Peronella japonica* [77], *Parechinus angulosus* [78]; starfish species *Patiriella exigua* [79, 80], *Asterias rubens* [79], and *Asterina minor* [81]; the sea cucumber *Holothuria glaberrima* [82]; the crinoids (feather star and sea lily) *O. japonicus* ([83], Tsurugaya, unpublished) and *Metacrinus rotundus *[84]; the brittle star *Stegophiura sladeni* [83]. In most of the cases, the sequences are partial, or only the homeobox of the genes are cloned, and the genomic organizations are not characterized. Therefore, comparison of the composition of Hox genes between or among echinoderm classes or species is possible only to a limited extent, and this may just be achieved after more genomic investigations. 

Originally, 10 Hox genes of *S. purpuratus* were obtained from various cloning methods [68], and by whole genome sequencing of *S. purpuratus*, the full set of 11 Hox genes, which are among the 96 homeobox transcription factors identified in the genome [85], was revealed. The Hox gene-containing genomic region was fully sequenced to determine the order of genes in the cluster [86]. 

From sequence similarity, Hox genes may be generally assigned to anterior (paralog groups (PG) 1-3), medial (PG 4-8), and posterior (PG 9-13) class genes. *S. purpuratus* possesses genes from all three classes, the anterior genes SpHox1-3, the medial genes SpHox 5-8, and the posterior genes SpHox9/10, 11/13a, 11/13b, and 11/13c. The posterior Hox genes are named as SpHox9/10, SpHox11/13a, b, and c genes, due to their similarity to the chordate PG9 and 10 genes or to PG11-13 genes [68]. No specific orthology relationships are apparent between the SpHox11/13a, b, and c genes and the chordate PG11-13 genes, but from phylogenetic analysis, these genes are shown to be included in a group of ambulacrarian (echinoderm + hemichordate) Hox11/13 genes, separated from chordates and other protostome genes, and thus could be regarded as a shared trait that supports the monophyly of ambulacrarians [87]. 

**Fig. (3):**
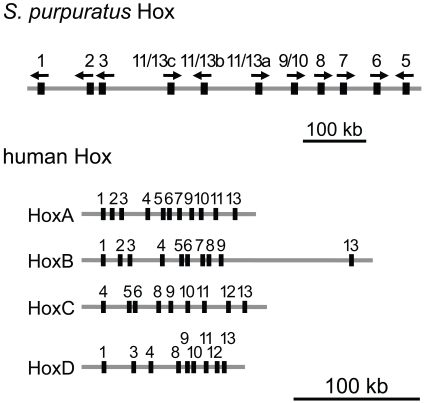
Gene order and positions of Hox genes in sea urchin (*S. purpuratus*) and human Hox clusters. The positions of the homeobox-coding exons are shown as boxes. The direction of transcription of *S. purpuratus* Hox genes are indicated above the boxes. All human Hox genes are transcribed in the direction from right to left. There are four Hox clusters (Hox A, B, C and D) in human. Note the difference in cluster sizes; *S. purpuratus* Hox genes are located in a region of ~600 kb, whereas most of the human Hox genes are included within ~100 kb.

It is now widely accepted that clusterization of Hox genes does not exist in all animals (see review by Duboule and Dolle [60]). For example, in urochordates (*C. intestinalis* and *Oikopleura dioica*) [88, 89], Hox genes are not all linked together or are even scattered on different chromosomes. The Hox genes of the sea urchin, on the other hand, are located in a single Hox cluster. However, this Hox genomic region shows a very unique structure in comparison to other animals, where the anterior Hox 1-3 genes are translocated next to the posterior Hox genes, and the gene order is Hox1, 2, 3, 11/13c, 11/13b, 11/13a, 9/10, 8, 7, 6, 5 (Fig. **3**). The sea urchin lacks the Hox4 gene, which is found in other echinoderms such as the crinoids and starfish, both of which have Hox 4 and 5 orthologs ([79, 83, 84] and Tsurugaya, unpublished). In addition to this apparent disorganization of the gene order on this chromosome, the orientation of the genes are not the same, opposed to the well-known vertebrate Hox cluster, in which genes are tightly positioned and all transcribed in the same direction. This indicates that there have been multiple steps of genomic rearrangement in the Hox region, including loss, duplication, translocation and inversion [86]. Nevertheless, middle and posterior group genes maintain the order. In addition, the size of the Hox cluster is much larger than the vertebrate counterparts (Fig. **3**).

As previously mentioned, expression is the key for the colinearity of the Hox cluster. Expression studies at the spatial or temporal levels, which are beyond the focus of this review, have been reported from a limited number of echinoderm species, *S. purpuratus* [64, 66, 90-92], *T. gratilla* [64, 69-71], *H. pulcherrimus* [73], *H. purpurescens* [93], *H. erythrogramma* [76], *H. tuberculata* [76], *P. angulosus* [78], *P. exigua* [94], and *M. rotundus* [84]. The reports that have investigated spatial colinearity in expression are those by Arenas-Mena *et al*. [90] and Hara* et al.* [84], of multiple Hox genes in *S. purpuratus* and *M. rotundus*, respectively. In *S. purpuratus*, a subset of Hox genes, Hox7, 8, 9/10, 11/13a and 11/13b, is sequentially expressed in the somatocoelar tissue of eight-arm larva, in a curved manner that parallels the curved gut with Hox7 at the larval mouth to 11/13b in the anal region [90]. In the case of *M. rotundus*, the presumptive somatocoel and later the left and right somatocoels express Hox genes in the order Hox5, 7, 8, 9/10 along the anterior-posterior axis [84]. Although, the gene order of crinoids is not known yet, at least for the five genes in sea urchin, the spatial expression pattern appears to coincide with the positions along the chromosome. Only Hox7 and 11/13b are reported to be expressed during development in *S. purpuratus* [64, 66, 90-92]. The expression of *S. purpuratus* Hox genes during the first 48 hours of development was reported [85], but no clear order of expression was detected, and Hox genes of *M. rotundus* do not show temporal colinearity [84], thus the presence of temporal colinearity is not proven so far in echinoderms. 

Ferrier and Holland [95] hypothesized a constraining force by mechanisms producing temporal colinearity that keeps Hox genes (or ParaHox genes, mentioned below) to be clustered, and when temporal colinearity becomes unnecessary for development, the clusters can be disorganized. On the other hand, despite rearrangements of the cluster, spatial colinearity is present in the portion of the cluster that remains generally organized. It should be noted that one of the genes that show spatial colinearity, namely Hox11/13b, is inverted, and therefore, it is quite interesting to find out whether there is a shared regulatory mechanism that controls expression of multiple genes, gene regulation functions or if its regulatory element is included in the inverted portion of the genome. Precise identification of gene regulatory elements is underway, and we are certainly at the stage, where we need to learn more about the Hox genomic region of species other than *S. purpuratus*.

The ParaHox cluster is a group of genes that is studied together with Hox genes in terms of comparison of genomic organization and evolution (reviewed in [96]). This cluster is considered to have arisen by a duplication of a proto-Hox gene cluster, and thus is an evolutionary sister of the Hox cluster. This cluster consists of three genes, gsx, xlox and cdx. However, the ParaHox cluster seems to be not strictly conserved evolutionarily, as loss of ParaHox genes and/or lack of linkage are observed in organisms such as *C. elegans* and *D. melanogaster* [95]. Amphioxus ParaHox genes exhibit spatial and temporal colinearity in expression, and are clustered in a genomic region within 35 kb [95, 97]. Three ParaHox genes, Sp-Gsx, Sp-lox, and Sp-Cdx, were identified from *S. purpuratus* [62]. These genes show spatial and temporal colinearity, although these genes are not linked to each other in the genome. This raises a question to whether the hypothesis of the correlation between clustering of ParaHox genes and temporal colinearity [95] is valid. As it is the case with Hox genes, to elucidate regulatory mechanisms of expression, data from more echinoderm species and comparisons are necessary.

## CONCLUSION: ECHINODERM GENOME COMPARISON AND FUTURE ISSUES

Sequencing of species other than *S. purpuratus* is currently progressing, for example, some BAC sequences for *L. variegatus* and *Asterina miniata* are available at the SpBase website. The whole-genome shotgun sequences of ~2.1x coverage for *Allocentrotus fragilis* and *S. franciscanus* were used, in addition to the *S. purpuratus* genome information, in a recent study [98]. *A. fragilis* and *S. purpuratus* are phylogenetically closely related, and *S. franciscanus*, which is used as the outgroup for comparison, is more distantly related. *S. purpuratus* and *S. franciscanus* inhabit throughout their lives intertidal and shallow waters, whereas *A. fragilis* spend their larval period in shallow waters, but as adults live in deeper waters. In this study, gene-coding sequences were extracted from the reads, and were investigated for protein evolution. The results showed that environmental adaptation functions as a driving force for divergence, since the proportion of genes under positive selection was higher in *A. fragilis* than in *S. purpuratus*. Moreover, increased positive selection was observed in genes expressed exclusively in adult somatic tissues than those in larvae or ovaries, reflecting the difference in the habitats of adults. These comparisons are only possible by collection and accumulation of genome sequence information. 

The features of the genome of one sea urchin species has been studied intensively, mostly in terms of gene content, but we need more information from other species for comparison, to look at the bigger picture. If we consider the unique features of echinoderms, there is even at the level of classes a surprising diversity, not only in morphology but for instance, the feeding adaptations. This leads us to believe that the genome of the echinoderms should harbor a vast amount of information on what kind of changes accumulated and how evolution has influenced the structure of the genome, which in turn, should reveal the basis of the specializations or inventions that echinoderms brought about during their evolution. Taking the sea urchin Hox cluster as an example, and comparing it with other phyla, there are rearrangements in the cluster that are quite amazing and intriguing, and this may be one of the reasons why echinoderms have characteristic morphologies. Thus, elucidation of lineage specific modifications in the genome, such as the change in synteny of genes, should undoubtedly tell us more about how echinoderms became what they are. 

## Figures and Tables

**Table 1.** List of Organism Groups with Genome Sequencing Projects, as of May, 2011 (http://www.ncbi.nlm.nih. gov/genomes/)

Type of organism (Phylum/Subphylum)	Number of entries
Placozoa	1
Porifera	1
Ctenophora	1
Cnidaria	5
Platyhelminthes	7
Nematoda	35
Rotifera	1
Mollusca	5
Priapulida	1
Arthropoda	84
Echinodermata	6
Hemichordata	1
Chordata	268
Total	416
